# TPI and GAPDH Interact with Rad9, Linking Glycolytic Enzymes to Cancer

**DOI:** 10.3390/ijms27125327

**Published:** 2026-06-12

**Authors:** Vivienne X. Y. Chua, Joyce M. X. Yip, Melody T. K. Cho, Sumi Z. Q. Lin, Rich Tan, Donna G. K. Lee, Kexin Dai, Teck K. Lim, Qingsong Lin, Rachel Lehming-Teo, Ophry Pines, Norbert Lehming

**Affiliations:** 1 Norbert Lehming, Department of Microbiology & Immunology and Cancer Programme at NUSMED, Yong Loo Lin School of Medicine, National University of Singapore, 5 Science Drive 2, Block MD4, Level 5, Singapore 117545, Singapore; chuavivienne927@gmail.com (V.X.Y.C.); joyceymx@gmail.com (J.M.X.Y.); melodychotsk@gmail.com (M.T.K.C.); sumweee.lin@gmail.com (S.Z.Q.L.); tanpinsoonrich@gmail.com (R.T.); donnaleegk@gmail.com (D.G.K.L.); daikexin912@gmail.com (K.D.); teo_shi_hui_rachel@schools.gov.sg (R.L.-T.); 2 Department of Biological Sciences, Faculty of Science, National University of Singapore, Singapore 117568, Singapore; dbslimtk@nus.edu.sg (T.K.L.); dbslinqs@nus.edu.sg (Q.L.); 3 Department of Microbiology and Molecular Genetics, IMRIC, Faculty of Medicine, Hebrew University of Jerusalem, Jerusalem 9190501, Israel

**Keywords:** aerobic glycolysis, checkpoint, DNA damage response, lysine lactylation, metabolic reprogramming

## Abstract

Cancer cells, like yeast, use fermentation despite the presence of oxygen, a phenomenon called aerobic glycolysis. The advantage is that it maintains many C-C bonds of glucose, allowing highly proliferating cells to produce the biomolecules that are necessary for cytokinesis. However, aerobic glycolysis is less energy-efficient than respiration, and it must operate at high frequency and produces large amounts of lactate, which modifies and stimulates DNA repair enzymes via lysine lactylation. This makes cancer cells resistant to radiotherapy, which requires a combination with chemotherapy using drugs that inhibit DNA repair. However, this converts healthy cells to cancer cells, indicating that research is still required regarding the relationship between glycolysis and cancer. Using yeast as a model, we discovered that the glycolytic enzymes TPI and GAPDH (Tpi1p and Tdh1-3p in yeast) interact with the DNA damage-dependent Checkpoint Rad9p (53BP1/BRCA1/MDC1 in humans). We propose that Tpi1p and Tdh1-3p override Rad9p, allowing cells with damaged DNA to proliferate. We isolated *tpi* and *gapdh* mutant strains that are deficient in DNA repair. While the *tpi* mutant strain has lower enzymatic activity, the *gapdh* mutant strains have normal enzymatic activity, confirming previous reports that GAPDH moonlights in the DNA damage response.

## 1. Introduction

“There is no Cure for this Disease.”—Hilaire Belloc [1].

“Tumours destroy man in a unique and appalling way, as flesh of his own flesh which has somehow been rendered proliferative, rampant, predatory and ungovernable. They are the most concrete and formidable of human maladies, yet despite more than 70 years of experimental study they remain the least understood.”—Francis Peyton Rous, tumour virologist, Nobel Lecture, 1966 [2].

Cancer is the principal cause of lethality worldwide, accounting for nearly 10 million deaths in 2022, or nearly one in six deaths [3,4]. Deregulated cellular metabolism is a hallmark of cancer that characterizes the cancer cell’s adjustments of energy metabolism for it to grow and divide [5]. In the presence of oxygen, normal, differentiated human cells inhibit fermentation and process glucose to pyruvate via glycolysis in energy-efficient mitochondrial oxidative phosphorylation, which is known as the Pasteur Effect [6,7]. An interesting observation that cancer cells carry out aerobic glycolysis was reported by Otto Warburg in the 1920s, who had coined the term glycolysis previously. In the presence of oxygen, the Warburg Effect, or aerobic glycolysis, occurs, converting glucose to lactate in the presence of oxygen [8]. Lactate was recently shown to post-translationally modify lysine residues of DNA repair factors like NSB1 [9], MRE11 [10], XRCC1 [11] and XLF [12], stimulating their activity (reviewed in [13]). Therefore, aerobic glycolysis makes cancer cells resistant to radiotherapy, and cancer treatment requires a combination with chemotherapy using drugs that inhibit DNA repair [14]. However, the combination can cause the conversion of healthy cells into cancer cells at a later point in time, which is one of the most fundamental paradoxes in modern oncology [15,16].

Aerobic glycolysis is also observed in yeast cells that are grown with high concentrations of glucose, where it is known as the Crabtree Effect [17]. In alcohol-producing yeast cells, aerobic glycolysis is triggered within a few seconds [18], while in tumour cells, the Warburg Effect requires protein expression and takes hours or even days to develop [19,20]. Aerobic glycolysis allows yeast and human cancer cells to generate energy in the form of ATP and NADH while maintaining C-C bonds that proliferating cells require for the synthesis of biomolecules [21]. However, per molecule of glucose, respiration produces approximately 36 ATP molecules, while aerobic glycolysis produces only two ATP molecules [21]. Therefore, aerobic glycolysis consumes much more glucose than respiration. We decided to use the budding yeast *Saccharomyces cerevisiae* as a model system to study the link between metabolism and cancer, as both yeast and human cancer cells are highly proliferative and rely on aerobic glycolysis to generate energy while maintaining C-C bonds to produce biomolecules like deoxyribose that are required to duplicate the DNA before cell division.

There are many known links between glycolysis and cancer, especially for triosephosphate isomerase (TPI), which catalyses the interconversion of dihydroxyacetone phosphate (DHAP) and glyceraldehyde-3-phosphate (GAP) in the glycolysis pathway [22], and for glyceraldehyde 3-phosphate dehydrogenase (GAPDH), which catalyses the conversion of GAP into glycerate-1,3-bisphosphate (1,3BPG) [23] (Supplementary Figure S1). TPI (Supplementary Figure S6) is highly expressed in many types of human tumours and is involved in the migration and invasion of cancer cells [24]. GAPDH (Supplementary Figure S9) plays a role in several cancer-related biological processes and has been reported to be commonly dysregulated in multiple cancer types [25]. Other metabolic enzymes and even metabolites moonlight in cancer as well [26]. Hexokinase in the first step of glycolysis is known to enter the nucleus and interact with nuclear proteins in acute myeloid leukemia (AML) [27]. Upon the occurrence of DNA double-strand breaks (DSB), GAPDH translocates into the nucleus, where it facilitates homologous recombination repair by stabilising RAD51 in an HDAC1-dependent manner [28]. According to the model proposed by Shi and co-workers, GAPDH disassociates HDAC1 from its inhibitor Maspin [28].

Rad9p is the prototypical DNA damage checkpoint, establishing the genetic regulation of the transient cell-cycle arrest that follows DNA damage [29]. Rad9p, a DNA damage sensor that binds DSBs, initiates the protein kinase signal transduction cascade as part of its role in the DNA damage response (DDR) by mediating phosphorylation of effector kinases like Chk1p and Rad53p [30,31]. Rad9p is required throughout the cell cycle as it has been shown to function at the transitions of G1/S, intra-S, and G2/M phases [32,33,34]. Rad9p is phosphorylated during normal progression of the cell cycle but becomes hyperphosphorylated by Mec1p and Tel1p in response to DNA damage [35,36]. Activated Rad9p then stimulates Mec1p phosphorylation of the effector kinases Chk1p and Rad53p [37,38,39]. Chk1p and Rad53p phosphorylation lead to processes associated with cellular arrest, such as transcriptional upregulation of DNA damage repair genes, transcriptional repression of cyclins, and stabilisation of replication forks [40,41]. Rad9p has two BRCT (BRCA1) domains that facilitate Rad9p–Rad9p interaction after DNA damage [42]. It also has a tandem Tudor domain that binds to double-stranded DNA [43]. *rad9* gene deletion strains are viable but exhibit sensitivity to X-ray and UV irradiation, fail to arrest in response to DNA damage, and are prone to chromosomal instability [44,45]. No single human ortholog has been identified; instead, in humans, there exists a family of proteins, which includes BRCA1 and 53BP1, that contain tandem BRCT domains [46,47].

### Hypothesis

Aerobic glycolysis, which proliferating cells use, is less energy-efficient than respiration. Therefore, glycolysis linked to fermentation (to alcohol in yeast cells and to lactic acid in human cancer cells) operates at a higher frequency than glycolysis linked to respiration, requiring higher glycolytic enzyme activity. We hypothesise that the glycolytic enzymes TPI1 and GAPDH override the DNA-damage checkpoint Rad9p in yeast cells and 53BP1/BRCA1/MDC1 in human cells. According to our model, high levels and activity of glycolytic enzymes inactivate Checkpoint Rad9p and allow cells with damaged DNA to proliferate.

## 2. Results and Discussion

### 2.1. Rad9p Interacts with Yeast Metabolic Enzymes

**Figure 1:**
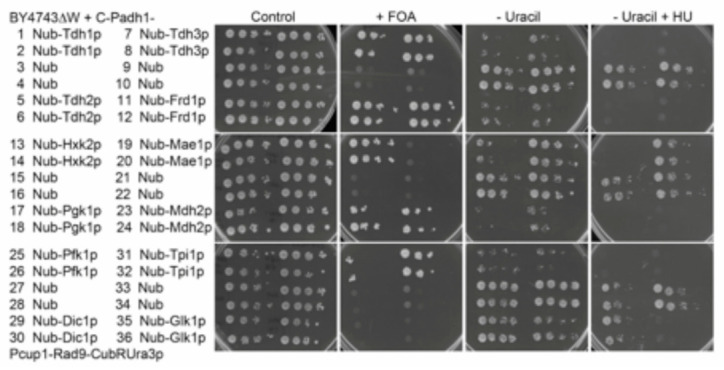
Rad9p interacted with metabolic enzymes in living yeast cells. Tenfold serial dilutions of cells expressing the indicated proteins were spotted onto the depicted plates and incubated at 28 °C for six days. Protein–protein interactions inside the living cells are revealed by growth on the FOA plates and by a lack of growth on plates lacking uracil. Each interaction presented in this figure was tested in at least one biological duplicate.

We tested the yeast DNA damage-dependent Checkpoint Rad9p for interaction with yeast metabolic enzymes in the Split-Ubiquitin (Split-Ub) assay. Figure 1 shows that Rad9p interacted with GAPDH (Tdh1p, Tdh2p & Tdh3p, lines 1, 2, 5, 6, 7, 8), Fumarate Reductase (Frd1p, lines 11, 12), Hexokinase 2 (Hxk2p, lines 13, 14), Phosphoglucokinase (Pgk1p, lines 17, 18), Cytosolic Malate Dehydrogenase 2 (Mdh2p, lines 23, 24), Phosphofructokinase 1 (Pfk1p, lines 25, 26) and triosephosphate isomerase (Tpi1p, lines 31, 32). When the cells were grown under conditions of DNA damage (+ hydroxyurea, HU), all these interactions became stronger, and Rad9p interacted additionally with Glucokinase 1 (Glk1p, lines 35, 36). No interaction was observed for Malic Enzyme (Mae1p, lines 19, 20), nor for the mitochondrial dicarboxylate carrier Dic1p (lines 29, 30).

### 2.2. A Tpi1 Mutant Strain Defective for the DDR Was Identified

**Figure 2:**
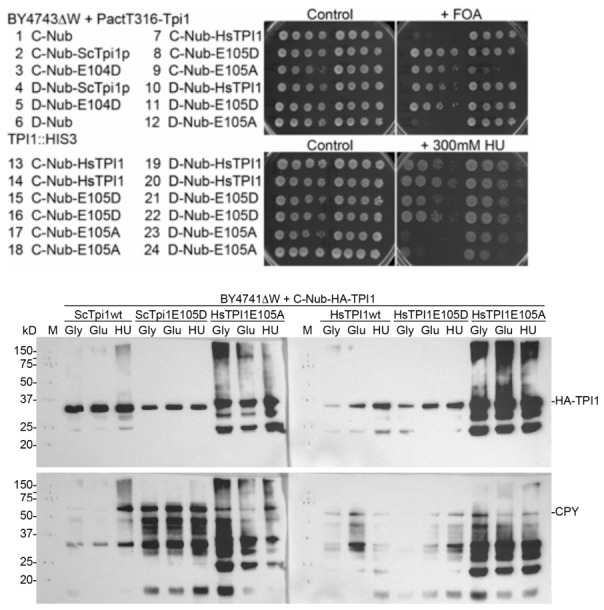
Yeast cells expressing human HsTPI1E105A in place of endogenous Tpi1p were viable but unable to grow under conditions of DNA damage. (**Left**) The *URA3*-marked plasmid PactT316-Tpi1 expressing endogenous Tpi1p was removed on the FOA plate (right top panel), and cells expressing Nub-HsTPI1 from single-copy (C) and multi-copy (D) plasmids were tenfold serially diluted and spotted onto 300 mM HU plates to test for their ability to confer sensitivity to HU. (**Right**) The HsTPI1E105A mutant protein was expressed at higher levels than the wild-type HsTPI1 protein. Yeast cells were grown to mid-log phase in glucose liquid media (Glu) and incubated for two hours in glycerol liquid media (Gly) or in glucose liquid media with 300 mM HU (HU). An equal number of cells were boiled in Sodium Dodecyl Sulfate (SDS) loading dye, and proteins were separated by SDS polyacrylamide gel electrophoresis (SDS-PAGE) and visualized with an anti-hemagglutinin (HA) antibody. As loading control, the membranes were re-probed with an anti-Carboxypeptidase Y (CPY) antibody. Each experiment presented in this figure was performed in at least biological duplicates.

The glycolytic enzymes Tpi1p, which interconverts DHAP and GAP, and GAPDH, which converts GAP to 1,3BPG, were chosen for further study. Human patients suffering from triosephosphate isomerase deficiency (TPID) express the HsTPI1E105D mutant protein [48]. TPID is an autosomal recessive multisystem disorder characterized by congenital hemolytic anemia, progressive neuromuscular dysfunction, susceptibility to bacterial infection, and cardiomyopathy [48]. While patients have lower TPI1 enzymatic activity in the serum, the mutation reduces the stability of TPI1 and does not alter its enzymatic activity. In order to determine if the conservative E105D mutation or the more severe E105A mutation would affect the DNA Damage Response (DDR) in yeast, we expressed Nub fusions of human HsTPI1 and its mutant derivatives under the control of the strong *ADH1* promoter from single-copy (C) and multi-copy (D) plasmids in yeast cells lacking the essential chromosomal *TPI1* gene that were kept alive by the *URA3*-marked plasmid PactT316-Tpi1, expressing Tpi1p from the *ACT1* promoter. The left panels of Figure 2 show that human HsTPI1 and its mutant derivatives were able to complement the yeast gene deletion strain and grow on the 5-fluoroorotic acid (FOA) plate, which counter-selects the PactT316-Tpi1p plasmid as Ura3p converts harmless FOA into toxic fluorouracil (lines 1 to 12). Cells expressing the HsTPI1E105A mutant protein from the single-copy vector displayed a growth phenotype (line 9). The figure further shows that the HsTPI1E105A mutant strain was sensitive to the DNA-damaging agent HU (lines 17, 18). Western Blots were performed to compare the expression levels of HsTPI1 with its mutant derivatives. The right panels of Figure 2 show that the E105A mutant protein was expressed at even higher levels than wild-type HsTPI1.

**Figure 3:**
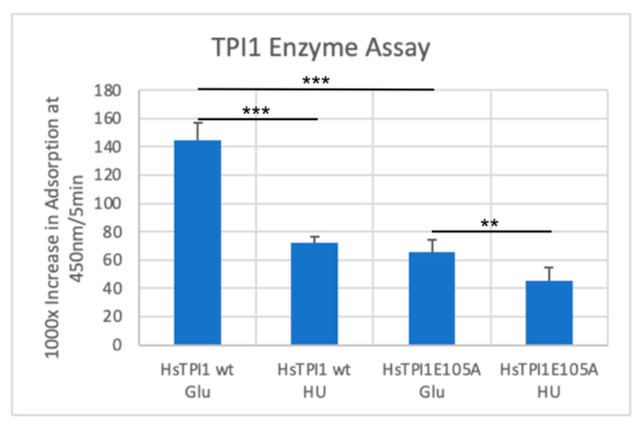
E105A mutation reduced the enzymatic activity of HsTPI1 twofold. Yeast cells expressing wild-type human HsTPI1 and the E105A mutant protein in place of yeast Tpi1p were grown to mid-log phase in glucose liquid media (Glu) and incubated with 300 mM HU for two hours (HU). Extracts were generated with bead beating, and the protein concentration was determined by Nanodrop. A total of 80 μg of extract was used for the TPI1 assay, and the increase in adsorption at 450 nm was measured for 5 min. We used two-sample *t*-tests (n = 3 experimental replicates, ** *p* ≤ 0.01, *** *p* ≤ 0.001), and the results are presented with error bars (SEM).

In order to determine if the E105A mutation reduced the enzymatic activity of HsTPI1, TPI commercial enzyme assays were performed with extracts from yeast cells expressing HA-tagged HsTPI1 and its mutant derivative in place of yeast Tpi1p. Figure 3 shows that the E105A mutation reduced the enzymatic activity of HsTPI1 by approximately two-fold. The figure further shows that exposure to DNA-damaging conditions for two hours reduced the enzymatic activity of HsTPI1 by approximately two-fold as well. Supplementary Figure S6 shows the location of the E105 residue relative to the catalytically active residues H96 and E166.

### 2.3. PTMs Specific for Conditions of DNA Damage Were Identified for Tpi1p and Tdh1p

**Figure 4:**
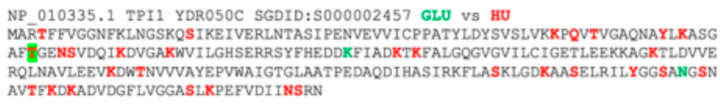
Tpi1p is post-translationally modified at thirty residues when cells are exposed to conditions of DNA damage. Residues shown in bold green were found to be modified specifically when the cells were grown under normal glucose conditions, and residues shown in bold red were found to be modified specifically when the cells were incubated for an additional two hours in glucose media containing 300 mM HU. A figure with the nature of these modifications and how often they had been identified can be found in Supplementary Figures S2 and S3. T75 was phosphorylated when the cells had been grown under normal glucose conditions and ubiquitinated when the cells had been exposed to 300 mM HU for an additional two hours.

Enzyme activity of TPI and GAPDH can be altered by post-translational modifications (PTMs), and we used mass spectrometry (MS) to identify PTMs that occur specifically when cells had been exposed for two hours to HU, which causes DSBs that are sensed by Rad9p. However, modified residues are much harder to identify by MS than unmodified residues. SDS-PAGE followed by excision of bands from the gel by size typically yields only about 1% of peptides from the protein of interest, while 99% originate from other proteins of similar size. A typical MS run identifies ~1000 peptides, and if just ten of them are from the protein of study, only one or two PTMs can be identified. Purification of proteins from protein extracts prior to SDS-PAGE, on the other hand, yields up to 99% of peptides from the protein of interest, and hundreds of PTMs are identified. Therefore, we purified Tpi1p fused to glutathione-S-transferase (GST) from yeast cells expressing GST-Tpi1p in place of endogenous Tpi1p that had been grown under normal glucose conditions and from yeast cells that had been exposed for an additional two hours to 300 mM HU. MS revealed that Tpi1p was specifically modified at thirty residues upon exposure to conditions of DNA damage (Figure 4). Our results for the TPI1 assay indicate that these modifications possibly reduce the enzymatic activity of Tpi1p.

**Figure 5:**
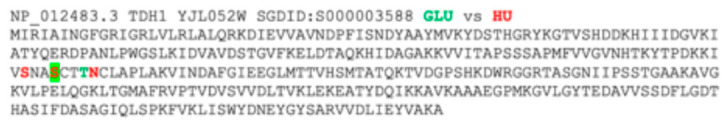
Tdh1p is post-translationally modified at three residues when cells are exposed to two hours of DNA damage. Residues shown in bold red were found to be modified specifically when the cells were grown in the presence of 300 mM HU for two hours, and residues shown in bold green were found to be modified specifically when the cells were grown under normal glucose conditions. S149 was methylated when the cells were grown under normal conditions and phosphorylated when the cells were exposed to 300 mM HU for two hours. Modifications were identified multiple times. See Supplementary Figure S4 for more details.

MS was also carried out for GST-Tdh1p purified from yeast cells expressing GST-Tdh1p in place of endogenous GAPDH that had been grown under normal glucose conditions versus yeast cells that had been exposed to conditions of DNA damage for an additional two hours. We found that Tdh1p was methylated at S149 and T152 when the cells were grown under normal conditions and phosphorylated at S146 and S149 and methylated at N153 when the cells had been exposed to 300 mM HU for two hours (Figure 5). These residues are close to the catalytically active C150, and S149 binds GAP, indicating that these modifications modify the enzymatic activity of GAPDH (Supplementary Figures S7 and S8).

**Figure 6:**
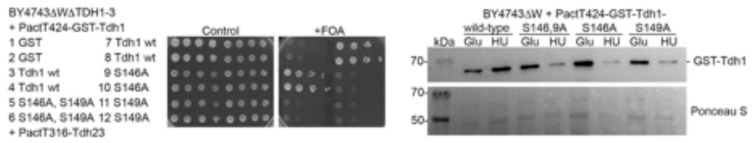
The Tdh1S146A and Tdh1S149A mutant proteins fail to complement a *TDH1-3* gene deletion strain. (**Left**) Yeast cells expressing the indicated GST fusions in place of endogenous Tdh1-3 proteins were tenfold serially diluted and spotted onto plates with and without FOA. Each growth assay was performed in at least biological duplicates. (**Right**) Yeast cells expressing the indicated GST fusions were grown to mid-log phase in glucose liquid media (Glu) and exposed to two hours of DNA damage (HU). Cells were boiled in SDS loading dye, and proteins were separated by SDS-PAGE. Proteins were blotted onto nitrocellulose, and GST fusions were visualized with the help of an anti-GST antibody. Each Western Blot was performed in biological triplicates. See Supplementary Figure S5 for the entire set.

In order to block post-translational modifications, S146 and S149 were mutated to alanine and expressed in yeast as GST fusions. Figure 6 shows that these mutant proteins were rapidly degraded when the cells were exposed for two hours to conditions of DNA damage. The figure further shows that the mutant proteins failed to complement a *TDH1-3* gene deletion strain that was kept alive with the *URA3*-marked plasmids PactT316-Tdh2/3, expressing Tdh2p and Tdh3p from the *ACT1* promoter, indicating that S146 and S149 are important for the enzymatic function of the essential Tdh1p.

### 2.4. Gapdh Mutant Strains Defective for the DDR Were Isolated

**Figure 7:**
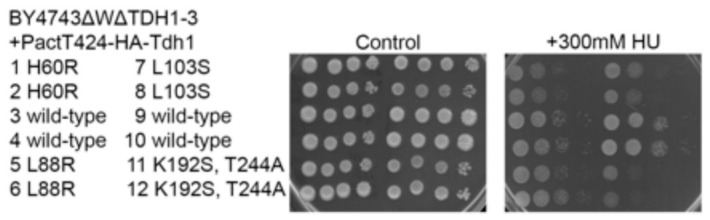
Random mutagenesis identified Tdh1 mutant proteins that confer sensitivity to HU. Tenfold serial dilutions of cells expressing the indicated proteins were spotted onto control plates and onto plates containing 300 mM HU. Each growth assay was performed in at least biological duplicates.

**Figure 8:**
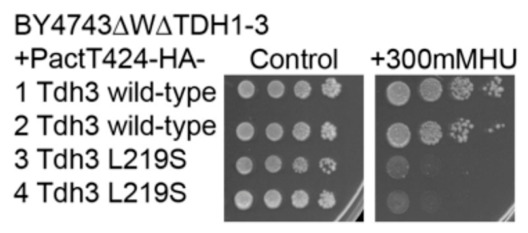
Random mutagenesis identified the Tdh3L219S mutant strain that is sensitive to HU. Tenfold serial dilutions of cells expressing the indicated proteins were spotted onto control plates and onto plates containing 300 mM HU. Each growth assay was performed in at least biological duplicates.

We used the *TDH1-3* gene deletion strain to generate GAPDH mutants that are sensitive to HU with the goal of studying the role of GAPDH in the DDR. Random PCR-based mutagenesis coupled with gap repair and plasmid shuffle on FOA plates, followed by replica plating, resulted in the Tdh1p mutant proteins H60R, L88R, L103S and K192S, T244A (Figure 7) and the Tdh3p mutant protein L219S (Figure 8). Subcloning revealed that only the T244A mutation caused sensitivity to HU and that the K192S mutation had no effect.

**Figure 9:**
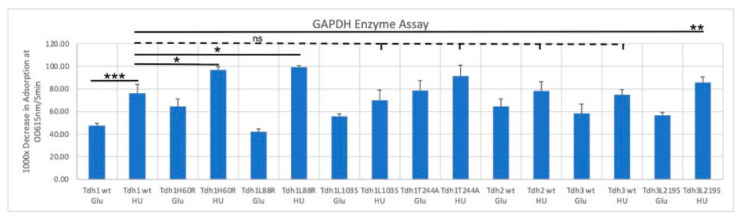
DNA damage conditions stimulated GAPDH activity, but the mutations had no effect on enzyme activity for GAP. Yeast cells expressing the indicated proteins were grown to mid-log phase in glucose liquid media (Glu) and treated with 300 mM HU for two hours (HU). Extracts were generated with bead beating, and the protein concentration was determined using nanodrop. A total of 80 µg of extract was used for the GAPDH assay, and the decrease in adsorption at 615 nm was measured for 5 min. Each enzyme assay was performed in at least experimental triplicates. Error bars represent the relative standard deviation of the mean. We used two-sample *t*-tests (n = 3 experimental replicates; ns = non-significant with *p* > 0.05, * *p* ≤ 0.05, ** *p* ≤ 0.01, *** *p* ≤ 0.001), and the results are presented with error bars (SEM).

In order to determine if the mutations reduced GAPDH enzymatic activity, GAPDH commercial enzyme assays were performed with extracts from yeast cells expressing GST-Tdh1/3p and their mutant derivatives in place of yeast Tdh1/3p. Figure 9 shows that exposure to DNA-damaging conditions for two hours increased the enzymatic activity of all fusions. Surprisingly, all mutant enzymes were fully active. One possible explanation is that GAPDH moonlights in the DDR with a function that is independent from its ability to interconvert GAP and 1,3BPG, i.e., by stabilising Rad51p [28].

### 2.5. A Yeast Mother’s Sacrifice

What is the incentive for a budding yeast cell with damaged DNA to proliferate? It has been proposed that yeast mothers sacrifice themselves for their daughters. The mothers keep all the damaged chromosomal DNA for themselves and pass the intact DNA to their newborns [49]. The same is true for ribosomes and even entire organelles like mitochondria [50]. Rather than sharing cellular damage evenly, the mother actively traps metabolic waste and extrachromosomal ribosomal DNA circles (ERCs) within her own cell body [51]. By keeping these aging factors, the mother ensures that the daughter can begin her life completely rejuvenated and capable of living a full life span [52]. At first glance, this sounds like an act of self-sacrifice; however, upon further reflection, it becomes clear that the budding yeast *Saccharomyces cerevisiae* has succeeded in fulfilling a dream humanity has dreamed for centuries: It has found the fountain of youth.

### 2.6. Limitations of This Study

We did not determine the domains of Rad9p that interact with Tpi1p and Tdh1p, but it would be interesting to study these interactions in more detail in the future. The TPI enzyme assay kit used in this study determines the activity of TPI for DHAP ⇔ GAP interconversion and not for the elimination reaction, where TPI converts DHAP to toxic methylglyoxal (MG). Therefore, the amount of MG generated by the TPI mutants was not measured. We determined the enzymatic activity of human TPI and GAPDH in extracts prepared from yeast cells expressing human TPI and GAPDH in place of endogenous yeast Tpi1p and yeast Tdh1-3p, which might differ from the activity of human TPI and human GAPDH expressed in human cells. Human cancer cells produce large amounts of lactate following the Warburg Effect. The recently described lactylation of lysine residues of DNA repair factors stimulates their activity, resulting in chemoresistance of cancer cells. We speculate that ethanol could react with, for example, glutamic acid side chains of yeast DNA repair factors, resulting in their esterification, which—according to our model—would stimulate those DNA repair factors as well.

## 3. Materials and Methods

### 3.1. Reagents

Yeast Lysis Buffer (0.1M Tris-HCl pH7.4; 50 mM KCl; 1 mM EDTA; 0.1% NP40), Glass Beads (Sigma G8772, St. Louis, MO, USA), Glutathione Sepharose (Amersham 17-5132-02, Little Chalfont, UK), Acrylamide (Bio Basic A800010, Markham, Canada), ammonium persulfate (Sigma A3678), TEMED (Sigma T22500), Ponceau S (Thermo Scientific A40000278, Hampton, NH, USA); Antibodies: anti-haemagglutinin (anti-HA, 12CA5, Roche 11 583 816 001), anti-glutathione-S-transferase (anti-GST, Novagen 71097-4, Madison, WI, USA), anti-Carboxypeptidase Y (anti-CPY, Life technologies A6428, Carlsbad, CA, USA), Goat anti-mouse-horseradish peroxidase (anti-mouse-HRP, BioRad 1706516, Hercules, CA, USA); Yeast Media: Adenine (Sigma A9126), Yeast Extract (Bio Basic G0961), Peptone (BD 211677), Glucose (Sigma G8270), Yeast Nitrogen Base (Bio Basic S507), Complete Supplement Mixture (MP 114500012-CF), Difco Agar (BD 214010); *E. coli* Media: Ampicillin (USB 11259), Chloramphenicol (Sigma C0378), Tryptone (Sigma T9410), NaOH (Merck 1.06498, Darmstadt, Germany); Kits: ECL Western Blotting Detection Reagent (Cytiva Prime RPN2232, Select RPN2235, Marlborough, MA, USA), *Taq* DNA Polymerase (Promega M3001, Madison, WI, USA), TPI enzyme assay (Sigma MAK274-1KT), GAPDH enzyme assay (Invitrogen AM1639, Carlsbad, CA, USA); Yeast strains: *BY4743ΔW TPI1::HIS3/TPI1::HIS3 + PactT316-Tpi1; BY4743ΔW TDH1::hisG/TDH1::hisG; TDH2::LEU2/TDH2::LEU2; TDH3::HIS3/TDH3::HIS3 + PactT316-Tdh2 + PactT316-Tdh3*; Chemicals: Agarose (Vivantis PC0701, Selangor, Malaysia), EDTA (Sigma E-5134), FOA (Fermentas R0812, Vilnius, Lithuania), Glycin (Fisher G/0800/60), HU (Bio Basic HB0528), Lithium-Acetate (Sigma L-4158), NaCl (Lab-Scan K2101T, Dublin, Ireland), and Polyethylenglycol 4000 (Merck 807490).

### 3.2. PCR-Based Random Mutagenesis of GAPDH

For each tube, 1 µL of template was added. A total of 49 µL of mix containing 10 µL of 5× buffer, 3 µL of 25 mM Magnesium Sulfate, 1 µL of nucleotides, 2 µL of forward primer, 2 µL of reverse primer, 30 µL of water, and 1 µL of *Taq* enzyme was distributed to each tube. The tubes were then mixed and spun down at 7000 rpm for 10 s. Finally, they were placed in the PCR machine with the program indicated below.
Step 1: 95.0 °C 0:01:00Step 2: 95.0 °C 0:00:30Step 3: 45.0 °C 0:00:30Step 4: 72.0 °C 0:01:00; 34× return to step 2Step 5: 72.0 °C 0:10:00Step 6: 4.0 °C ∞

### 3.3. Generation of the Yeast Strain Expressing TPI1 Derivatives in Place of Endogenous Tpi1p

The tryptophan auxotrophic *BY4741/2/3ΔW* strains have been described previously [53]. *BY4741ΔW* and *BY4742ΔW* cells were transformed with the *URA3*-marked single-copy plasmid PactT316-Tpi1, which expressed Tpi1p under the control of the strong constitutive *ACT1* promoter/terminator cassette. Next, the *TPI1* gene was replaced with the *HIS3* gene via homologous recombination, and the strains were mated to generate the diploid *BY4743ΔW TPI1::HIS3/TPI1::HIS3 + PactT316-Tpi1* strain. The strain was either transformed with *LEU2*-marked single- and multi-copy plasmids expressing Nub-HA-Tpi1p wild-type and mutant or Nub-HA-HsTPI1 wild-type and mutant under the control of the strong constitutive *ADH1* promoter/terminator cassette or the empty vector control, and with the *TRP1*-marked multi-copy vector PactT424-GST-TPI1 expressing GST-Tp1p under the control of the strong constitutive *ACT1* promoter/terminator cassette or the empty vector control. Plasmid shuffle was performed on plates containing 5-fluoroorotic acid (FOA), as Ura3p, Orotidine-5′-phosphate decarboxylase, converts FOA into toxic fluorouracil (Figure 2).

### 3.4. Generation of the Yeast Strain Expressing Tdh Derivatives in Place of Endogenous Tdh1-3

The *TDH1* gene of *BY4741ΔW* and *BY4742ΔW* was replaced with the *TRP1* gene. Next, the *TRP1* marker was recovered with the help of the *TR-hisG-URA3-hisG-P1* cassette of NKY1009 [54], and the *URA3* marker was recovered on an FOA plate. Subsequently, the *TDH2* gene was replaced with the *LEU2* gene, and the *BY4741ΔW* strain was transformed with PactT316-Tdh2, while the *BY4742ΔW* strain, which was generated later, was transformed with PactT316-Tdh3. Finally, the *TDH3* gene was replaced with *HIS3,* and the strains were mated to generate *BY4743ΔW TDH1::hisG/TDH1::hisG; TDH2::LEU2/TDH2::LEU2; TDH3::HIS3/TDH3::HIS3 + PactT316-Tdh2 + PactT316-Tdh3*, named *BY4743ΔWΔTDH1-3 + PactT316-Tdh23* in this study. The strain was transformed with the *TRP1*-marked plasmids PactT424-GST-Tdh123 wild-type and mutants, and PactT316-Tdh23 was shuffled out on an FOA plate.

### 3.5. Yeast Breaking for Enzyme Assay

Cells were resuspended and transferred into a screw cap tube before 100 µL of appropriate buffer (KDalert buffer for GAPDH assay, yeast lysis buffer for TPI assay) was added. Afterward, glass beads of equal volume to the cells were added to the same screw cap tube. Next, the cells were broken in the bead beater at 6000 rpm, 3 × 20s , 10 s interval. After each cycle, the tubes were placed into an ice box for 3 min, and breaking and cooling were repeated 3 times. Subsequently, the cells were placed into a 4 °C centrifuge and spun down at 15,000 rpm for 10 min. Finally, the supernatant was transferred into a new Eppendorf tube.

### 3.6. TPI Enzyme Assay

The TPI Enzyme Assay Kit (Amersham) was used to determine the TPI enzyme activity. To a cuvette, 44 µL of TPI Assay Buffer, 2 µL of TPI Enzyme Mix, 2 µL of TPI Developer and 2 µL of TPI Substrate were added. On top of that, a volume of yeast extract, depending on the protein concentration measured by Nanodrop, was added. The cuvette was then topped up to 100 µL with TPI Assay Buffer. A total of 900 µL of sterile water was added to the cuvette and mixed. After that, the cuvette was placed into the spectrophotometer to measure TPI enzyme activity at 450 nm for 5 min in 30 s intervals. In the absence of substrate, no activity was detected.

### 3.7. GAPDH Enzyme Assay

The KDalert^TM^ GAPDH Assay Kit (Invitrogen) was used to measure GAPDH enzyme activity. To a cuvette, 88.8 µL of solution A, 0.68 µL of solution B and 0.47 µL of solution C were added. Solutions A, B and C contain the required substrates: glyceraldehyde-3-phosphate (GAP), phosphate ions and NAD^+^ to measure GAPDH enzyme activity. A total of 20 µL of yeast protein extract was added and mixed with 900 µL of water in the cuvette. After that, the cuvette was placed into the spectrophotometer to measure GAPDH enzyme activity at 615 nm for 5 min in 30 s intervals. In the absence of substrate, no activity was detected.

### 3.8. Mass Spectrometry

GST-Tpi1p and GST-Tdh1p were expressed in yeast cells in place of the endogenous proteins, and 50 mL liquid glucose cultures were grown in the absence and presence of 300 mM HU. Protein extracts were generated as described above, and GST fusion proteins were isolated with gluthathione beads and further purified using SDS-PAGE. The gel was stained with InstantBlue Coomassie Protein Stain. The bands corresponding to GST-Tpi1p and GST-Tdh1p were cut out and in-gel trypsinated. The tryptic peptides were subjected to liquid chromatography–mass spectrometry (LC-MS) analysis using an Eksigent nanolC Ultra and ChiPLC-nanoflex (Eksigent, Dublin, CA, USA) in trap-elute configuration, with a 200 μm × 0.5 mm trap column and a 75 μm × 150 mm analytical column. Both trap and analytical columns were made of ChromXP C18-CL, 3 μm (Eksigent). Peptides were separated by a gradient formed by 2% ACN, 0.1% FA (mobile phase A) and 98% ACN, 0.1% FA (mobile phase B): 5% to 7% of mobile phase B in 0.1 min, 7% to 30% of mobile phase B in 10 min, 30% to 60% of mobile phase B in 4min, 60% to 90% of mobile phase B in 1 min, 90% to 90% of mobile phase B in 5 min, 90% to 5% of mobile phase B in 1 min and 5% to 5% of mobile phase B in 10 min, at a flow rate of 300 nl/min. MS analysis was performed on a TripleTOF 5600 system (AB SCIEX, Foster City, CA, USA) in Information Dependent Mode. MS spectra were acquired across the mass range of 400–1250 m/z in high-resolution mode (>30,000) using a 250 ms accumulation time per spectrum. A maximum of 10 precursors per cycle were chosen for fragmentation from each MS spectrum, with 100 ms of minimum accumulation time for each precursor and dynamic exclusion for 8 s. Tandem mass spectra were recorded in high-sensitivity mode (resolution >15,000) with rolling collision energy on. Peptide identification and the detection of post-translational modifications were carried out using ProteinPilot 5.0.3.0 software Revision 5313 (AB SCIEX) using the Paragon database search algorithm (5.0.3.0.5312) against a protein sequence database (6128 entries) of the yeast *Saccharomyces cerevisiae*.

### 3.9. The Split-Ubiquitin (Split-Ub) Assay

**Figure 10:**
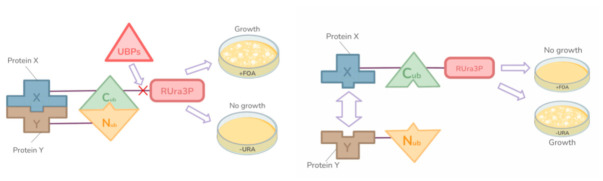
The conditional protein degradation design of the Split-Ub System. The Nub construct consists of a protein of interest fused to the N-terminal half of ubiquitin, and the Cub construct is made of a protein of interest fused to the C-terminal of ubiquitin extended by the RUra3 protein, which is Orotidine-5′-phosphate decarboxylase whose first amino acid has been replaced with an arginine (R). (**Left**) If proteins X and Y interact inside the cell, Nub and Cub fold upon each other and are recognized by the ubiquitin-specific proteases (UBPs). RUra3p is cleaved off and so rapidly degraded that the cells become uracil auxotrophic and resistant to FOA. (**Right**) If proteins X and Y do not interact inside the cell, RUra3p is not cleaved off and not degraded. Therefore, the cells remain uracil prototrophic and sensitive to FOA.

The Nub construct consists of a protein of interest fused to the N-terminal half of ubiquitin, while the Cub construct is made of a protein of interest fused to the C-terminus of ubiquitin extended by the RUra3 protein, which is Orotidine-5′-phosphate decarboxylase whose first amino acid has been replaced with an arginine (R) [55]. If the two proteins of interest interact inside the living cell, the Cub attached to Protein Y will be in close proximity with the Nub that was fused to protein X (Figure 10, left panel). This results in the formation of a native-like Ubiquitin moiety that will be recognized by ubiquitin-specific proteases (UBPs), leading to UBPs cleaving the bond between Cub and RUra3p. Free RUra3p will be rapidly degraded by 26S proteasomes due to the N-end rule [56]. As a result, there will no longer be Orotidine-5′-phosphate decarboxylase in the cells. When these cells are plated onto an FOA plate, Ura3p is not present to convert FOA into toxic fluorouracil, allowing the cells to survive. However, when these cells are plated onto plates lacking uracil (-URA), there will be no Ura3p present to synthesise uracil required for RNA synthesis, thereby resulting in a lack of growth. If the two proteins of interest do not interact inside the living cell, the Cub that is attached to Protein X and the Nub fused to protein Y will not be in close proximity (Figure 10, right panel). As such, a full native-like ubiquitin moiety will not be formed, and the UBPs will not cleave the peptide bond between the Cub and RUra3p. As a result, RUra3p will not be degraded and will still be present inside the yeast cell. When these cells are plated onto FOA plates (+FOA), Ura3p will be present to convert FOA into toxic fluorouracil, resulting in the cells dying. On the other hand, when these cells are plated onto plates lacking uracil (-URA), the cells will be able to make uracil, allowing the cells to grow. The Split-Ub assay has been validated in many scientific publications [57,58,59,60,61,62,63,64,65,66,67,68].

## 4. Conclusions

We hypothesised that the glycolytic enzymes TPI and GAPDH override the DNA damage-dependent Checkpoint Rad9p in yeast and 53BP1/BRCA1/MDC1 in human cells when cells are grown with glucose as the main carbon source. Yeast mothers give their daughters a step up by acting as biological dustbins, which means mothers divide such that they keep the damaged DNA for themselves and pass the intact DNA to their daughters. In the presence of glucose, TPI and GAPDH allow cells with damaged DNA to proliferate and their rejuvenated daughters to consume the glucose before it is eaten by the competition. We found that TPI and GAPDH interacted with Rad9p in the Split-Ub assay, and we isolated yeast strains expressing mutant derivatives of TPI and GAPDH in place of the endogenous enzymes that were unable to grow under conditions of DNA damage. For TPI, we found that the human TPI1E105A mutant protein, which confers sensitivity to HU, displayed reduced enzymatic activity, implicating the enzymatic activity of TPI in its ability to override Rad9p. For GAPDH, on the other hand, we also identified GAPDH mutant proteins that conferred sensitivity to HU; however, those mutant proteins were enzymatically fully active, possibly reflecting previous reports that GAPDH moonlights in the DDR with a function that is unrelated to its enzymatic activity, as it was proposed that GAPDH is a chaperone that prevents proteolytic degradation of HDAC1.

## Data Availability

The original contributions presented in this study are included in the article/Supplementary Materials. Further inquiries can be directed to the corresponding author.

## Supplementary Materials

The following supporting information can be downloaded at: https://www.mdpi.com/article/10.3390/ijms27125327/s1.
