# Supplementary material for: TPI and GAPDH Interact with Rad9, Linking Glycolytic Enzymes to Cancer
Source: Int J Mol Sci. 2026 Jun 12;27(12):5327. doi: 10.3390/ijms27125327 (PMC13300414; doi:10.3390/ijms27125327)
Supplement: Supplementary file 1 [file ijms-27-05327-s001.zip › ijms-4319715-supplementary.pdf]

SUPPLEMENTARY

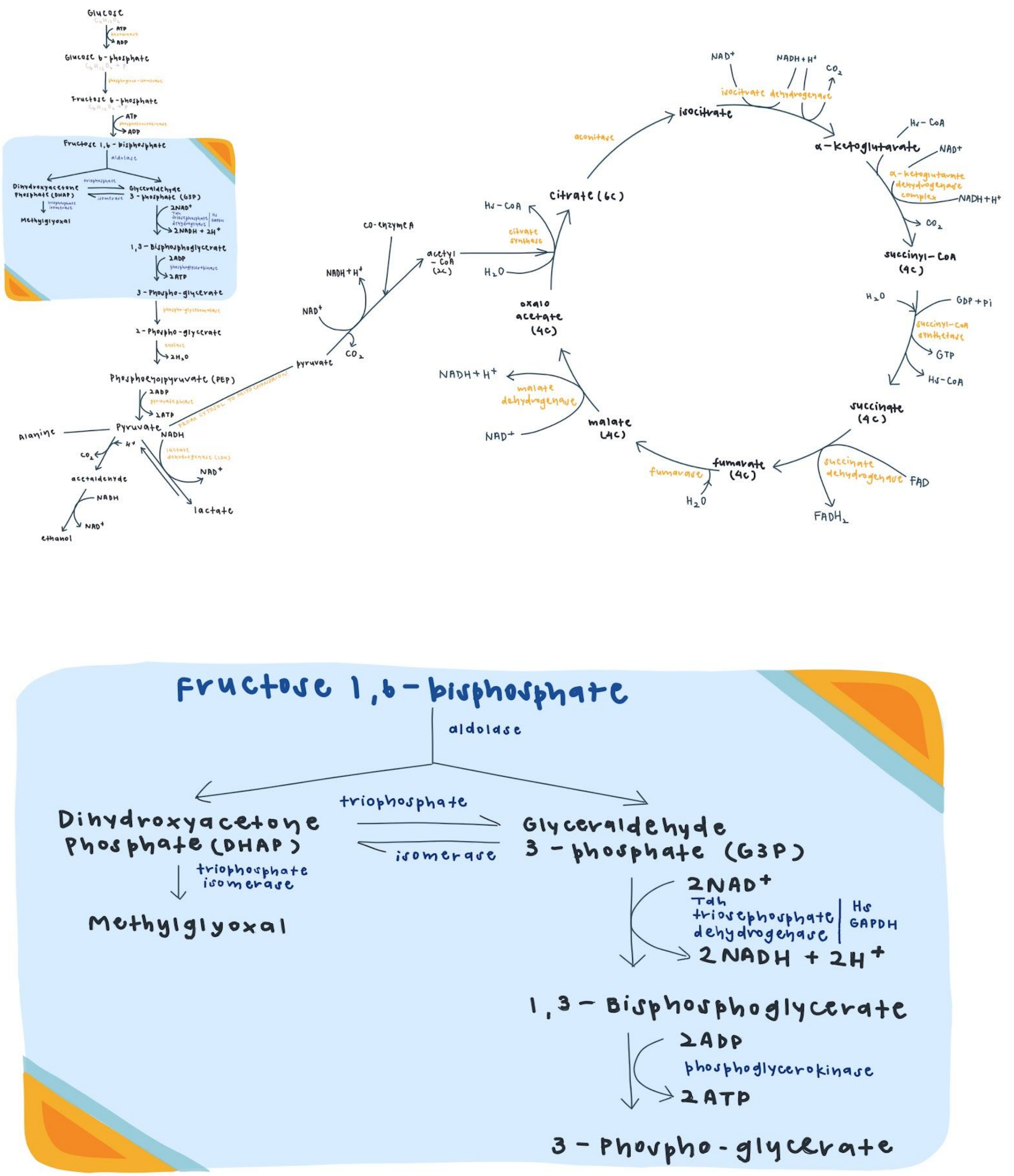

Supplementary Figure S1. Glycolysis and Krebs cycle. Highlighted are the location of metabolic enzymes TPI and GAPDH in the 4th and 5th step of glycolysis.

```
NP_010335.1 TPI1 YDR050C SGDID:S000002457 GLU vs HU
MARTFFVGGNFKLNGSKQSIKEIVERLNTASIPENVEVVICPPATYLDYSVSLVKKPQVTVGAQNAYLKASG
AFTGENSVDQIKDVGAKWVILGHSERRSYFHEDDKFIADKTKFALGQGVGVILCIGETLEEKKAGKTLDVVE
RQLNAVLEEVKDWTNVVVAYEPVWAIGTGLAATPEDAQDIHASIRKFLASKLGDKAASELRILYGGSANGSN
AVTFKDKADVDGFLVGGASLKPEFVDIINSRN
```

**GST-Tpi1 (Glu)**	**232 Peptides**
**Peptide**	**[Protein Modification]**
SGAFTGENSVDQIKDVGAK	T75:Phosphorylation
HEDDKFIADKTK	K107:Succinylation
ILYGGSANGSNAVTFK	N213:HexNAcylation

Supplementary Figure S2. Protein modifications obtained for GST-Tpi1 purified from cells grown in liquid media under normal, non-DNA damaging conditions (Glu).

**GST-Tpi1 (HU)**	**657 Peptides**
**Peptide**	**Protein Modification**
RTFFVGGNFK	T4:Ubiquitin
SIKEIVER	S19:Ubiquitin
KPQVTVGAQNAYLK	K56:Methylation
KPQVTVGAQNAYLK	K56:Ubiquitin
QVTVGAQNAYLKASGAFTGENSVDQIK	Q58:Ub;T60:Phospho;Y67:Methylation
KPQVTVGAQNAYLK	Y67:Phosphorylation;K69:Ubiquitin
KPQVTVGAQNAYLKASG	K69:Myristoylation;S71:Phosphorylatio
KASGAFTGENSVDQIKDVGAK	K69:Dimethylation;S71:Phosphorylation
KASGAFTGENSVDQIKDVGAK	K69:Acetylation
KASGAFTGENSVDQIKDVGAK	K69:Dimethylation
KASGAFTGENSVDQIKDVGAK	K69:Methylation
KASGAFTGENSVDQIKDVGAK	K69:Succinylation
KASGAFTGENSVDQIKDVGAK	K69:Ubiquitin
KASGAFTGENSVDQIKDVGAK	S71:Ubiquitin
ASGAFTGENSVDQIKDVGAK	T75:Ubiquitin;S79:Ubiquitin
ASGAFTGENSVDQIK	T75:Ubiquitin
GAFTGENSVDQIKDVGAK	T75:Ubiquitin
AFTGENSVDQIKDVGAK	T75:Ubiquitin;S79:Ubiquitin
GAFTGENSVDQIKDVGAK	N78:Ubiquitin
ASGAFTGENSVDQIKDVGAK	S79:Phosphorylation;K89:Ubiquitin
ASGAFTGENSVDQI	S79:Ubiquitin
ASGAFTGENSVDQIK	S79:Ubiquitin
ASGAFTGENSV	S79:Ubiquitin
NSVDQIKDVGAK	S79:Ubiquitin
ASGAFTGENSVDQIKDVGAK	K84:Methylation
ASGAFTGENSVDQIK	K84:Succinylation
SYFHEDDKFIADKTK	K112:Acetylation;K114:Ubiquitin
KTLDVVER	K138:Succinylation
QLNAVLEEVK	K155:Succinylation
QLNAVLEEVKDWT	T158:Ubiquitin
LASKLGDKAASELRILYGGSANGSNAVTFK	S194;S202:Phosphorylation
ASKLGDKAASELRILYGGSANGSNAVTFK	S194:Ubiquitin;K199:Succinylation
ASKLGDKAASELRILYGGSANGSNAVTFK	S202:Ubiquitin
KAASELR	K199:Acetylation
RILYGGSANGSNAVTFK	Y208:Hexose
ILYGGSANGSNAVTFK	Y208:Thiophosphorylation;S211:Ub
ILYGGSANGSNAVTFK	Y208:Ubiquitin
ILYGGSANGSNAVTFKDK	S211:Ubiquitin;S215:Ubiquitin
ILYGGSANGSNAVTFKD	T219:Ubiquitin
ILYGGSANGSNAVTFK	T219:Ubiquitin;K221:Ubiquitin
ILYGGSANGSNAVTF	T219:Ubiquitin
DKADVDGFLVGGASLKPEFVDIINSR	K223:Ubiquitin
KADVDGFLVGGASLKPEFVDIINSR	K223:Succinylation
DKADVDGFLVGGASLKPEFVDIINSR	S235:Ubiquitin;K237:Acetylation
LVGGASLKPEFVDIINSR	S235:Ubiquitin
SLKPEFVDIINSR	S235:Ubiquitin
DKADVDGFLVGGASLKP	K237:Ubiquitin
LKPEFVDIINSR	K237:Methylation
DKADVDGFLVGGASLKPEFVDIINS	N245:Ubiquitin
DKADVDGFLVGGASLKPEFVDIINSR	S246:Ubiquitin
GFLVGGASLKPEFVDIINSR	S246:Ubiquitin
GGASLKPEFVDIINSR	S246:Ubiquitin

Supplementary Figure S3: Protein modifications obtained for GST-Tpi1 purified from cells grown in liquid media under conditions of DNA damage (HU).

NP_012483.3 TDH1 YJL052W SGDID:S000003588 **GLU** vs **HU**
MIRIAINGFGRIGRLVLRLALQRKDIEVVAVNDPFISNDYAAYMVKYDSTHGRYKGTVSHDDKHIIIDGVKI
ATYQERDPANLPWGSLKIDVAVDSTGVFKELDTAQKHIDAGAKKVVITAPSSSAPMFVVGVNHTKYTPDKKI
V**S**NA**S**CT**TN**CLAPLAKVINDAFGIEEGLMTTVHSMTATQKTVDGPSHKDWRGGRTASGNIIPSSTGAAKAVG
KVLPELQGKLTGMAFRVPTVDVSVVDLTVKLEKEATYDQIKKAVKAAAEGPMKGVLGYTEDAVVSSDFLGDT
HASIFDASAGIQLSPKFVKLISWYDNEYGYSARVVDLIEYVAKA

**GST-Tdh1 (Glu)**	**708 Peptides**
**Peptide**	**[Protein Modification]**
IVSNA**S**CTTN	[Methyl(S)@149]
IVSNASCT**T**N	[Methyl(T)@152]

**GST-Tdh1 (HU)**	**333 Peptides**
**Peptide**	**[Protein Modification]**
IV**S**NASCTTNCLAPLAK	[Phospho(S)@146]
IVSNA**S**CTTNCLAPLAK	[Phospho(S)@149]
IVSNASCTT**N**CLAPLAK	[Methyl(N)@153]

Supplementary Figure S4: Protein modifications obtained for GST-Tdh1p purified from cells grown in liquid media under normal conditions (Glu) and under conditions of DNA damage (HU).

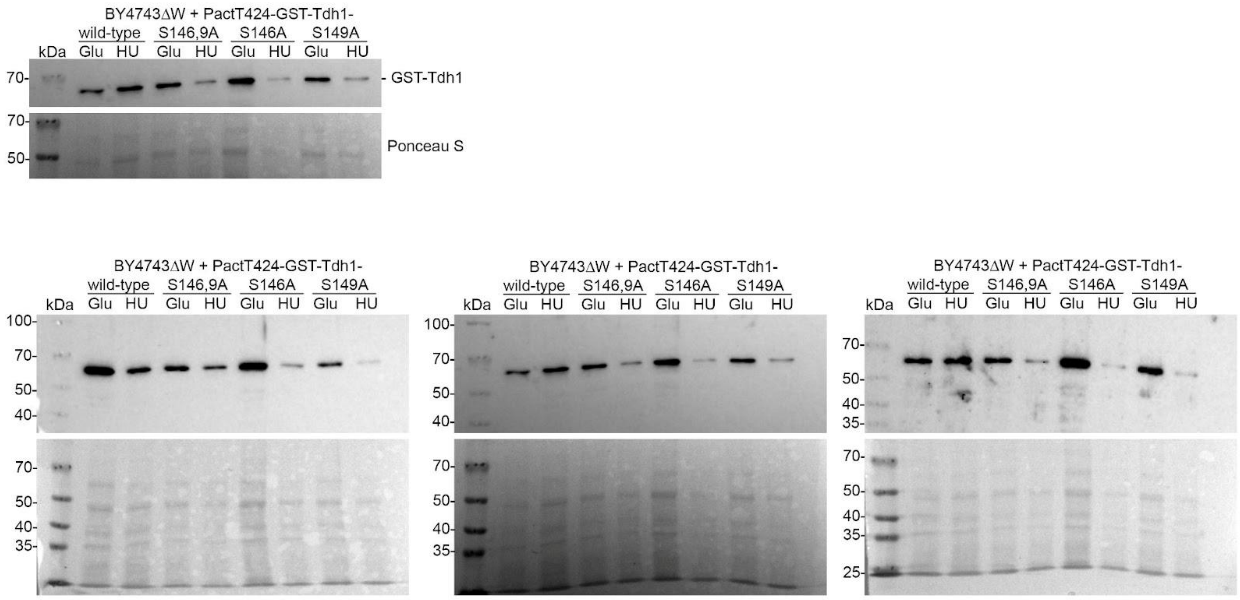

Supplementary Figure S5: GST-Tdh1 wt and mutants are expressed.

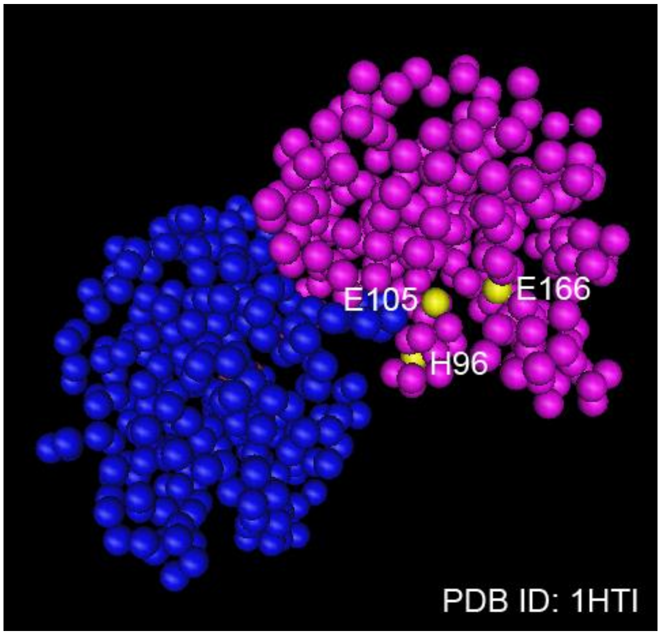

Supplementary Figure S6. Crystal Structure of human TPI1. The structural data of human HsTPI1 (Mande, 1994) were downloaded from NCBI and opened with Cn3D. The positions of E105 and of the catalytically active H96 and E166 were indicated with PhotoShop.

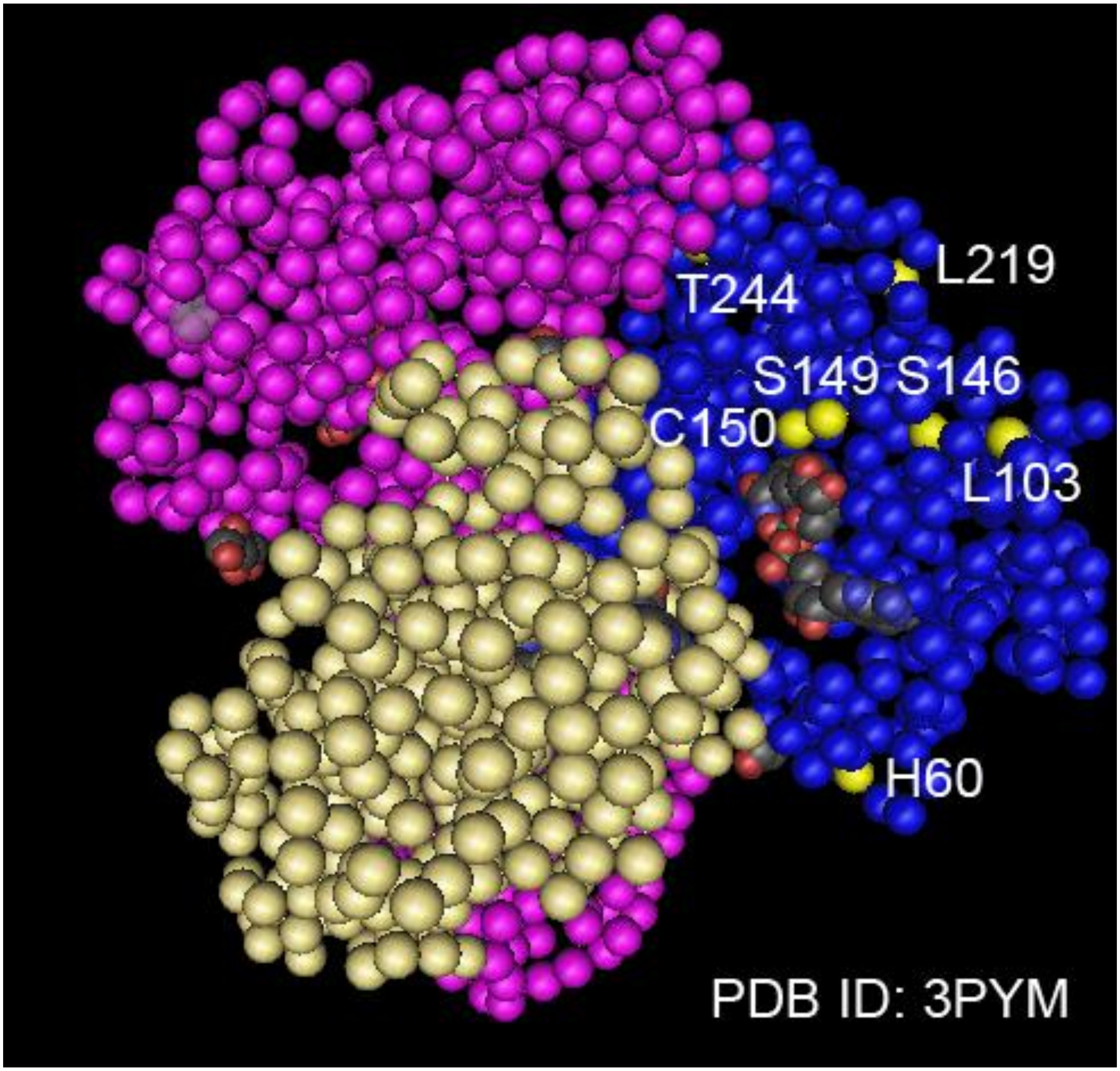

Supplementary Figure S7. Crystal Structure of yeast Tdh3 (GAPDH). The structural data of yeast Tdh3 were downloaded from NCBI and opened with Cn3D. The positions of the catalytically active C150 and of H60, L103, S146, S149, L219 and T244 were indicated with PhotoShop.

```
glyceraldehyde-3-phosphate dehydrogenase (phosphorylating) TDH1 vs TDH2 vs TDH3
[Saccharomyces cerevisiae S288C]
Sequence ID: NP_012483.3 vs NP_012542.1 vs NP_011708.3 Length: 332
                                                                     H60R
TDH1  1    MIRIAINGFGRIGRLVLRLALQRKDIEVVAVNDPFISNDYAAYMVKYDSTHGRYKGTVSH  60
           M+R+AINGFGRIGRLV+R+ALQRK++EVVA+NDPFISNDY+AYM KYDSTHGRY G VSH
TDH2  1    MVRVAINGFGRIGRLVMRIALQRKNVEVVALNDPFISNDYSAYMFKYDSTHGRYAGEVSH  60
           MVRVAINGFGRIGRLVMRIAL R NVEVVALNDPFI+NDY+AYMFKYDSTHGRYAGEVSH
TDH3  1    MVRVAINGFGRIGRLVMRIALSRPNVEVVALNDPFITNDYAAYMFKYDSTHGRYAGEVSH  60
                                      L88R           L103S
TDH1  61   DDKHIIIDGVKIATYQERDPANLPWGSLKIDVAVDSTGVFKELDTAQKHIDAGAKKVVIT  120
           DDKHII+DG KIAT+QERDPANLPW SL ID+A+DSTGVFKELDTAQKHIDAGAKKVVIT
TDH2  61   DDKHIIVDGHKIATFQERDPANLPWASLNIDIAIDSTGVFKELDTAQKHIDAGAKKVVIT  120
           DDKHIIVDG KIAT+QERDPANLPW S N+DIAIDSTGVFKELDTAQKHIDAGAKKVVIT
TDH3  61   DDKHIIVDGKKIATYQERDPANLPWGSSNVDIAIDSTGVFKELDTAQKHIDAGAKKVVIT  120
                            S146A S149A C150
TDH1  121  APSSSAPMFVVGVNHTKYTPDKKIVSNASCTTNCLAPLAKVINDAFGIEEGLMTTVHSMT  180
           APSS+APMFV+GVN  KYT D KIVSNASCTTNCLAPLAKVINDAFGIEEGLMTTVHSMT
TDH2  121  APSSTAPMFVMGVNEEKYTSDLKIVSNASCTTNCLAPLAKVINDAFGIEEGLMTTVHSMT  180
           APSSTAPMFVMGVNEEKYTSDLKIVSNASCTTNCLAPLAKVINDAFGIEEGLMTTVHS+T
TDH3  121  APSSTAPMFVMGVNEEKYTSDLKIVSNASCTTNCLAPLAKVINDAFGIEEGLMTTVHSLT  180
                                                     L219S
TDH1  181  ATQKTVDGPSHKDWRGGRTASGNIIPSSTGAAKAVGKVLPELQGKLTGMAFRVPTVDVSV  240
           ATQKTVDGPSHKDWRGGRTASGNIIPSSTGAAKAVGKVLPELQGKLTGMAFRVPTVDVSV
TDH2  181  ATQKTVDGPSHKDWRGGRTASGNIIPSSTGAAKAVGKVLPELQGKLTGMAFRVPTVDVSV  240
           ATQKTVDGPSHKDWRGGRTASGNIIPSSTGAAKAVGKVLPELQGKLTGMAFRVPTVDVSV
TDH3  181  ATQKTVDGPSHKDWRGGRTASGNIIPSSTGAAKAVGKVLPELQGKLTGMAFRVPTVDVSV  240
              T244E
TDH1  241  VDLTVKLEKEATYDQIKKAVKAAAEGPMKGVLGYTEDAVVSSDFLGDTHASIFDASAGIQ  300
           VDLTVKL KE TYD+IKK VKAAAEG +KGVLGYTEDAVVSSDFLGD+++SIFDA+AGIQ
TDH2  241  VDLTVKLNKETTYDEIKKVVKAAAEGKLKGVLGYTEDAVVSSDFLGDSNSSIFDAAAGIQ  300
           VDLTVKLNKETTYDEIKKVVKAAAEGKLKGVLGYTEDAVVSSDFLGDS+SSIFDA+AGIQ
TDH3  241  VDLTVKLNKETTYDEIKKVVKAAAEGKLKGVLGYTEDAVVSSDFLGDSHSSIFDASAGIQ  300

TDH1  301  LSPKFVKLISWYDNEYGYSARVVDLIEYVAKA  332
           LSPKFVKL+SWYDNEYGYS RVVDL+E+VAKA
TDH2  301  LSPKFVKLVSWYDNEYGYSTRVVDLVEHVAKA  332
           LSPKFVKLVSWYDNEYGYSTRVVDLVEHVAKA
TDH3  301  LSPKFVKLVSWYDNEYGYSTRVVDLVEHVAKA  332
```

Supplementary Figure S8. Protein sequence alignment of yeast Tdh1p, Tdh2p and Tdh3p. BLAST was performed at NCBI and the positions of the catalytically active C150 and of the mutations identified in this project are indicated.

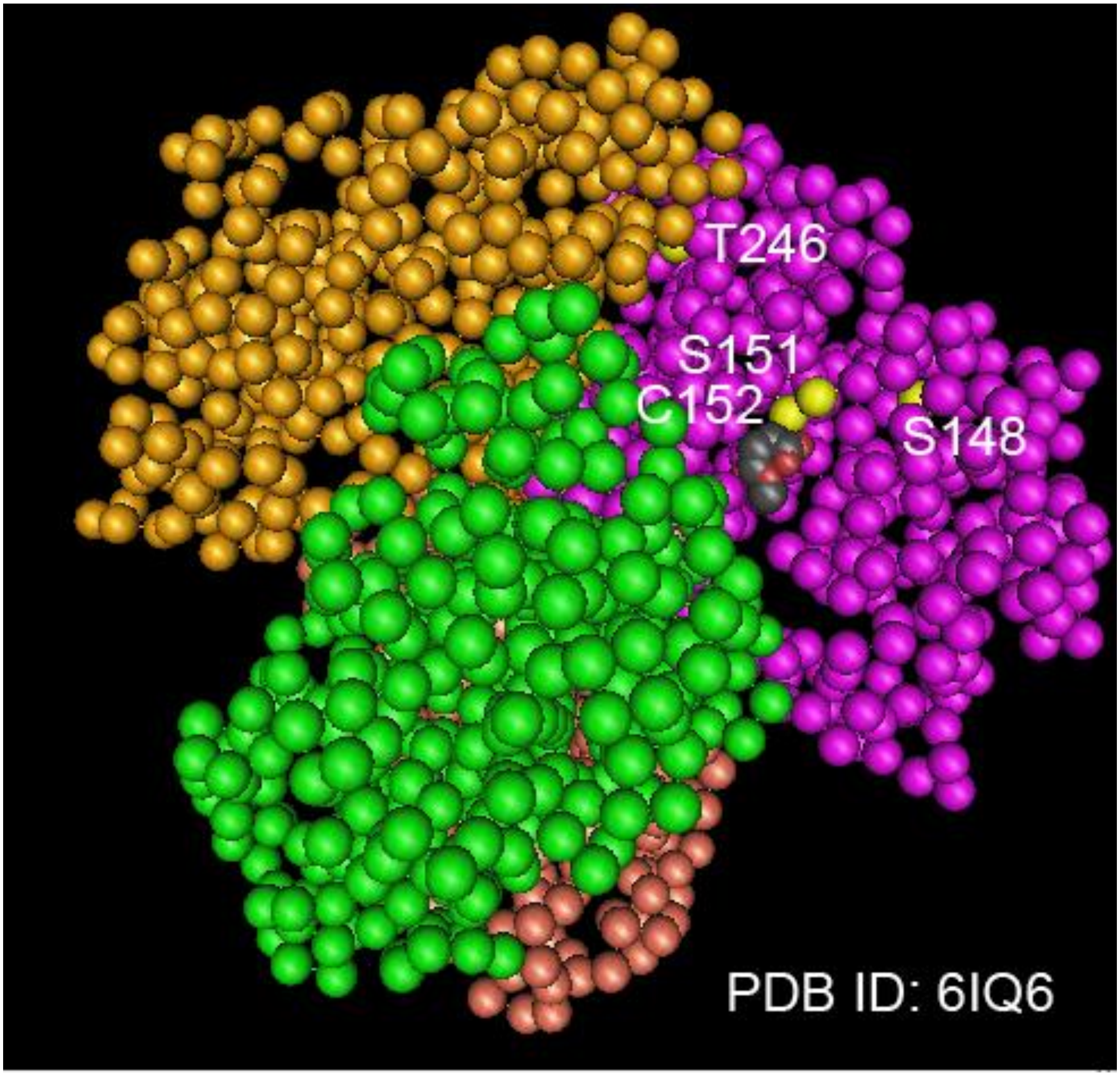

Supplementary Figure S9: Crystal Structure of human GAPDH. The structural data of human GAPDH (Park, 2019) were downloaded from NCBI and opened with Cn3D. The positions of the catalytically active C152 and of S148, S151 and T246 were indicated with PhotoShop.
